# Longitudinal Changes in QTc Interval in Cancer Patients After the Initiation of Systemic Therapy: Analysis of the ONCOECHO Study Cohort

**DOI:** 10.3390/cancers18182936

**Published:** 2026-09-10

**Authors:** Maciej Siński, M. Zaborska-Dworak, P. Sobieraj, J. Lewandowski, B. Zaborska, K. Mizia-Stec, Z. Gąsior, J. D. Kasprzak, I. Kowalik, P. Stachowiak, P. Gościniak

**Affiliations:** 1 Department of Internal Medicine, Hypertension and Vascular Diseases, Medical University of Warsaw, 02-097 Warsaw, Poland; maciej.sinski@wum.edu.pl (M.S.); jacek.lewandowski@wum.edu.pl (J.L.); 2 Department of Cardiology, Grochowski Hospital, Centre of Postgraduate Medical School, 04-073 Warsaw, Poland; bzaborska@cmkp.edu.pl; 3 First Department of Cardiology, Medical University of Silesia, 40-635 Katowice, Poland; kmiziastec@gmail.com; 4 Department of Cardiology, Faculty of Health Sciences, Medical University of Silesia, 40-635 Katowice, Poland; zgasior@sum.edu.pl; 5 1st Department of Cardiology, Medical University of Lodz, 91-347 Lodz, Poland; kasprzak@ptkardio.pl; 6 Department of Coronary Artery Disease and Cardiac Rehabilitation, National Institute of Cardiology, 04-628 Warsaw, Poland; ikowalik@ikard.pl; 7 Department of Cardiology, Pomeranian Medical University, 70-111 Szczecin, Poland; stachowiak@sedimed.pl; 8 Cardiometabolism and Cardio-Oncology Laboratory, Department of Endocrinology and Internal Medicine, Pomeranian Medical University, 71-252 Szczecin, Poland

**Keywords:** QTc interval, cardio-oncology, chemotherapy, electrocardiography, cardiotoxicity

## Abstract

**Background/Objectives:** Anticancer therapies are associated with a prolongation of QT interval of electrocardiogram (ECG), increasing the risk of arrhythmias. To evaluate this risk in cardiovascular disease-free subjects with cancer, ECG recorded at baseline, months 3, 6 and 12 from 291 participants from the ONCOECHO database were analyzed. **Methods:** Both Fridericia (QTcF), considered as standard, and Bazett (QTcB) formulas were used to calculate corrected QT (QTc). **Results:** The study cohort consisted of 61.8% subjects with breast cancer, 26.5% with hematologic malignancies, 7.3% with colorectal cancer and 4.4% of patients with kidney cancer. At baseline, six patients had a QTcF ≥ 460 ms and one ≥ 500 ms. QTcF values differed significantly across time points (*p* = 0.0125), with a significant increase between baseline and month 6 (400.9 ± 29 ms vs. 409.6 ± 25 ms, *p* = 0.0075). Use of the Bazett formula resulted in 5.49 (95% CI 3.28–9.19) higher odds of identification of QTc ≥ 460 ms than with the Fridericia rule. A LASSO logistic regression model identified age, QTcF at baseline and month 3 to increase odds of QTcF ≥ 460 ms, whereas higher ejection fraction was associated with decreased odds. **Conclusions:** Analysis shows that cancer patients receiving cardiotoxic therapy are at relatively low risk of QTc prolongation, but choosing the appropriate QTc correction formula is essential.

## Simple Summary

Some cancer treatments can affect the heart’s electrical activity and prolong the QT interval on an electrocardiogram, which may increase the risk of dangerous heart rhythm disturbances. However, the frequency and pattern of these changes in patients without known cardiovascular disease are not fully understood. This study investigated how the QT interval changes during the first year after starting systemic cancer treatment and explored which patients may be more likely to develop clinically relevant prolongation. The analysis included 291 patients with newly diagnosed cancer who underwent electrocardiographic assessment before treatment and during follow-up. The findings showed a modest overall increase in the corrected QT interval, while clinically important prolongation and serious ventricular arrhythmias were uncommon. Older age and higher QT measurements early in treatment were associated with greater risk. The results support individualized electrocardiographic monitoring and careful selection of the method used to assess QT duration.

## 1. Introduction

The advances in the treatment of cancer have led to significant improvements in cancer-related mortality [1]. Almost half of patients diagnosed with cancer are now alive 10 years after diagnosis, and this remarkable improvement in cancer survivorship is, in large part, due to the increased anti-tumor effects of a rapidly broadening range of available cancer therapies. However, these advancements in treatment have been accompanied by numerous cardiovascular side effects: cardiovascular disease is a major cause of morbidity and mortality in cancer survivors [2], and the cardiotoxic potential of anticancer drugs may compromise the long-term outlook of affected patients [3]. New treatment options, which are responsible for the mortality improvement, may evoke QTc interval prolongation [4]. QTc interval prolongation represents a concern in cardio-oncology, as it impairs myocardial repolarization and increases the risk of fatal arrhythmias. Drug-induced QTc prolongation is linked to an increased risk of TdP, a specific form of polymorphic ventricular tachycardia that causes rapid hemodynamic compromise and may degenerate into ventricular fibrillation; TdP causes syncope and carries a high risk of arrhythmic sudden cardiac death [5]. This atypical tachycardia, named from the French for “twisting of the points”, is characterized by oscillations of the points or peaks around the main axis of the ECG and, although often self-limited, can degenerate into ventricular fibrillation, thus leading to sudden cardiac death [6]. Although the relationship between QTc and the risk of TdP is complex and influenced by dynamic variables, a QTc > 500 ms is generally accepted to be associated with a clinically significant risk of ventricular arrhythmia and sudden cardiac death [5]; indeed, QTc intervals ≥ 500 ms increase the risk of cardiac events such as syncope, aborted cardiac arrest, TdP or sudden cardiac death approximately tenfold. Accordingly, a QTc > 500 ms and an increase from baseline of >60 ms are considered of particular concern, since torsades de pointes rarely occurs when QTc is below 500 ms [7]. Most drug-induced QTc prolongation is thought to be caused by direct blockade of the pore-forming subunit of the cardiac potassium channel that carries the rapid delayed rectifier current, the major determinant of repolarization in ventricular myocytes [5]. The hERG gene encodes these pore-forming subunits of the IKr channel [8], and drug-induced interference with IKr is the most common cause of acquired long QT syndrome and TdP; accordingly, QT prolongation and interaction with hERG channels have become surrogate markers of cardiotoxicity attracting increasing regulatory attention [9,10]. Nevertheless, the extent to which key QTc modulators such as heart rate, age, gender, and electrolyte status could be factored into risk assessments and treatment protocols remains unclear.

Cancer patients have more chance to acquire QTc prolongation due to factors such as polypharmacy encompassing anticancer drugs, supportive therapies, and comorbidity management [11,12]. Furthermore, underlying cancer disease and prevalent gastrointestinal toxicity—including nausea, vomiting, and diarrhea—often induce severe electrolyte imbalances, amplifying this hazard [12]. Prior retrospective evaluations of antineoplastic agents, like tyrosine kinase inhibitors, have documented different QTc prolongation rates from 5.8% to 28% [13]. However, the prevalence and clinical implications of QTc prolongation in a diverse, unselected cohort of incident cancer patients receiving varied chemotherapy remain incompletely defined [12,13,14]. This study evaluated the prevalence of QTc prolongation among newly diagnosed cancer patients starting chemotherapy. The aim of this study was to assess the prevalence of QTc prolongation in patients with newly diagnosed cancer initiating systemic treatment enrolled into an ONCOECHO observational study [15,16].

## 2. Materials and Methods

### 2.1. Study Design and Overview of the ONCOECHO Study

This study utilized data from the multicenter ONCOECHO study database [15,16]. ONCOECHO was a prospective, observational, multicenter study conducted between 2012 and 2014 in reference cardiovascular units. The primary aim of the ONCOECHO study was to investigate the cardiovascular consequences, particularly cancer therapy-related cardiac dysfunction, of systemic anticancer treatment, including chemotherapy and targeted therapy, in patients with newly diagnosed malignancies.

Patients were recruited at the beginning of systemic anticancer treatment, including chemotherapy, targeted therapies, and immunotherapies. The study protocol included a standardized clinical assessment, resting 12-lead electrocardiography (ECG), transthoracic echocardiography (TTE), and laboratory testing. Standardized medical history and physical examination were performed, with particular attention to oncological characteristics, cardiac history, cardiovascular comorbidities, and concomitant medications. Blood pressure, heart rate, height, and weight were recorded. Laboratory assessment included complete blood count, lipid profile, fasting glucose, serum creatinine, and natriuretic peptides (NT-proBNP or BNP).

### 2.2. Study Population and Cohort Selection

For the present analysis, baseline ECGs from 343 patients enrolled in the ONCOECHO database were subjected to a comprehensive review. The ONCOECHO population comprised consecutive patients screened before planned systemic treatment for newly diagnosed cancer. The parent cohort included patients with breast cancer, hematological malignancies, colorectal cancer, and kidney cancer, as previously described.

Patients were excluded if they had any of the following: baseline left ventricular ejection fraction (LVEF) < 55%; regional left ventricular (LV) wall motion abnormalities at rest; LV wall thickness > 13 mm corresponding to LV hypertrophy; moderate or severe valvular heart disease; or previous treatment with anthracyclines or radiotherapy. These criteria were applied to exclude patients with pre-existing structural or functional cardiac abnormalities that could interfere with the assessment of cardiovascular effects of anticancer treatment.

Following application of these exclusion criteria, 291 patients were included in the present baseline analysis.

Baseline cardiovascular status was assessed before initiation of systemic anticancer treatment. The standardized clinical assessment included cardiac history, cardiovascular comorbidities, and concomitant medications. Physical examination included measurement of blood pressure, heart rate, height, and weight. A standard 12-lead resting ECG was performed to assess heart rate and rhythm, QT interval duration, arrhythmias, conduction abnormalities, and ST-segment and T-wave abnormalities. TTE was performed to assess LV systolic and diastolic function, right ventricular size and function, valvular status, and the presence of pericardial effusion.

Follow-up resting ECGs were available at 3 months (*n* = 242), 6 months (*n* = 167), and 12 months (*n* = 128) after initiation of systemic anticancer treatment. These serial measurements enabled an assessment of changes in QTc over time and evaluation of the temporal pattern of QTc prolongation during treatment.

The ONCOECHO study was approved by the relevant Bioethical Committees, and all participants provided written informed consent before enrollment.

### 2.3. QTc Interval Assessment and Analysis

QTc intervals were primarily calculated using the Fridericia correction formula, considered the standard correction method in the present analysis (QTcF = QT/RR^1/3). A secondary analysis was performed using the Bazett formula (QTcB = QT/RR^1/2), as this correction is frequently used for automated ECG analysis by ECG devices and remains commonly used in outpatient clinical practice.

The following thresholds for QTc prolongation were applied based on commonly used definitions [17] and ESC guidelines [18]. QTc ≥ 460 ms was considered increased irrespective of sex. QTc ≥ 480 ms was considered clinically relevant according to ESC guidelines and indicative of the need for cardio-oncology consultation and follow-up. QTc ≥ 500 ms was considered a threshold potentially requiring withdrawal or modification of anticancer treatment and increased ECG surveillance [18]. An absolute increase in QTc of ≥60 ms from baseline was considered clinically important. In addition, an increase in QTc of ≥10 ms from baseline was considered a threshold suggesting potential proarrhythmic risk.

### 2.4. Statistical Analysis

All continuous variables were expressed as mean ± standard deviation or median with interquartile range as appropriate for their distribution, while categorical variables were presented as frequencies. Distribution of the data was assessed using Shapiro–Wilk test and visually assessed using q-q plots. A *p*-value < 0.05 was considered statistically significant. Differences in QTc interval measurements over time and between patient subgroups were analyzed using repeated measures ANOVA (normally distributed variables) or Friedman’s test (non-normally distributed variables) with appropriate post hoc tests. Incidence of QTc prolongation using different methods of calculation (QTc ≥ 460 ms; QTc ≥ 480 ms; QTc ≥ 500 ms; an increase in QTc ≥ 60 ms from baseline) was compared using Fisher exact test. Odds ratios (ORs) comparing Bazett (QTcB) versus Fridericia (QTcF) corrections for three QTc thresholds were calculated. Values were presented as ORs with 95% confidence intervals (CI). ORs were computed as (a/b)/(c/d), where a/b are QTcB cases/non-cases and c/d are QTcF cases/non-cases at the same time point. 95% CI from log(OR) ± 1.96 × SE with SE = sqrt(1/a + 1/b + 1/c + 1/d). Haldane–Anscombe correction (+0.5 to each cell) was applied when any cell count equaled zero (for month 12 for ≥480 and ≥500 ms). The association between the QTc interval, administered medications, and cancer type was visualized using heatmap. A LASSO logistic regression model was applied to identify independent predictors of QTcF increase above a threshold of 460 ms at month 6, with coefficient shrinkage used to retain only the most relevant variables. As potential predictors, age, sex, type of cancer, QTcF (at baseline and month 3), EF (at baseline and month 3), and E/e′ (at baseline and month 3) were considered.

## 3. Results

### 3.1. Study Participants’ Characteristics and QTc Prolongation

**Figure 1:**
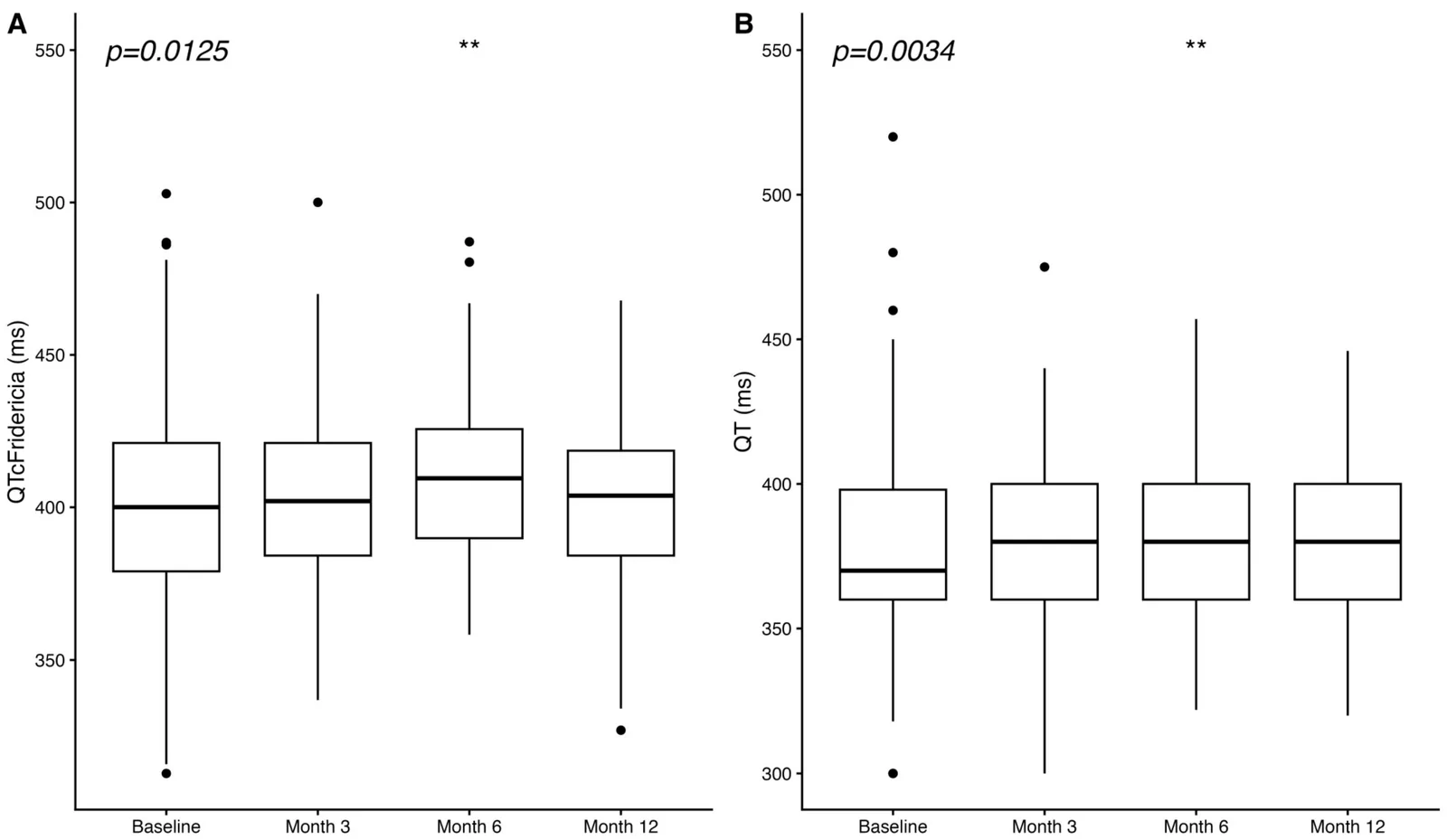
Boxplots of QT and QTcF intervals over time. Panel (**A**)—Boxplots of QTc values calculated using Fridericia’s formula. Boxes represent the interquartile range (IQR), the central line indicates the median, and whiskers represent the minimum and maximum values. *p* = 0.0075 for the post hoc comparison between baseline and month 6. Panel (**B**)—Boxplots of QT values. Boxes represent the interquartile range (IQR), the central line indicates the median, and whiskers represent the minimum and maximum values. *p* = 0.0024 for the post hoc comparison between baseline and month 6. ** indicates *p* < 0.01.

The study cohort consisted of 212 (61.8%) subjects with breast cancer, 91 (26.5%) with hematologic malignancies, 25 (7.3%) with colorectal cancer, and 15 (4.4%) of patients with kidney cancer. Of the 343 patients initially screened, 52 met the predefined exclusion criteria; therefore, 291 patients were included in the final baseline analysis. Mean age of patients was 55.5 ± 12.8 years) and mean BMI was 26.4 ± 4.8 kg/m^2^. A total of 78.7% (270) of patients were females. At baseline, the mean QTcF was 400.9 ± 29.6 ms, and one subject had a QTcF ≥ 500 ms (520 ms), while six patients had a QTcF ≥ 460 ms. QTcF values differed significantly across time points (*p* = 0.0125), with a significant increase between baseline and month 6 (400.9 ± 29 ms vs. 409.6 ± 25 ms, *p* = 0.0075) (Figure 1, panel A). Uncorrected QT interval analysis revealed significant differences (*p* = 0.0034), with a significant increase at month 6 (*p* = 0.0024) (Figure 1, panel B).

**Figure 2:**
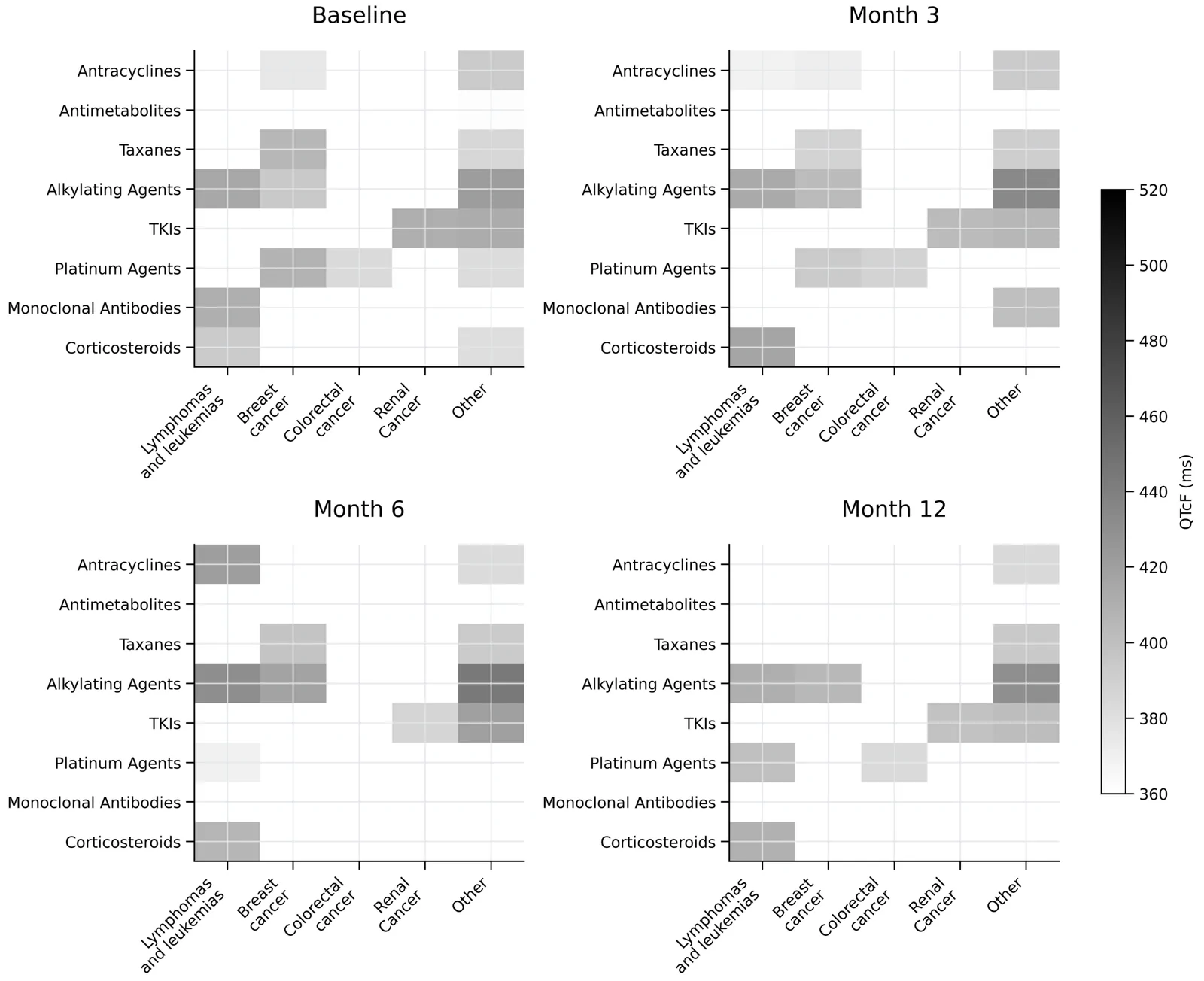
Heatmap of mean QTcF interval across treatment groups/cancer groups at baseline, 3 months, 6 months, and 12 months. Four heatmaps display the average QTcF interval (ms) for major chemotherapy-related treatment groups over time. QTcF values are shown on a unified grayscale scale ranging from 250 to 550 ms. Treatment categories include anthracyclines, taxanes, alkylating agents, antimetabolites, platinum agents, tyrosine kinase inhibitors (TKIs), monoclonal antibodies, and corticosteroids. Cancer classifications were grouped as breast cancer, lymphomas and leukemias (combined category of lymphoproliferative malignancies and chronic myeloid leukemia), colorectal cancer, renal cell carcinoma, and others. This figure illustrates the longitudinal variation in QTcF intervals across treatment modalities and cancer types.

At month 6, seventy-one patients showed a QTcF prolongation of more than 10 ms from baseline; however, only three patients experienced a prolongation exceeding 60 ms from baseline. Of these, six patients had a QTcF exceeding 460 ms, and two subjects’ QTcF exceeded 480 ms. After 12 months, the mean QTcF was 413 ± 30 ms, and 41 patients exhibited a QTcF prolongation of more than 10 ms. Among these, only two patients had a QTcF exceeding 460 ms, and none had a QTcF longer than 480 and 500 ms. Interestingly, QTcF did not increase continuously in individual subjects; different patients exhibited their longest QTcF at different time points. Furthermore, no clinically important ventricular arrhythmias were observed. The heatmap analysis (Figure 2) demonstrated marked heterogeneity in QTcF across treatment classes, diagnostic groups, and time points.

### 3.2. QTc Calculation with Bazett in Comparison to Fridericia Formula

Baseline QTc was higher when the Bazett instead of the Fridericia rule was used: 416.3 ± 34.7 ms compared to 400.9 ± 29.6 ms, *p* < 0.0001.

Among 828 ECG recordings, prolongation of QTc ≥ 460 ms was present in 90 (10.9%) cases in comparison to 18 (2.2%), respectively, prolongation of QTC ≥ 480 ms and ≥500 ms was present in 28 (3.4%) and 11 (1.3%) of tracings when the Bazett formula was used in comparison to the Fridericia formula: 7 (0.8%) and 2 (0.2%).

A detailed comparison of QTc prolongation with thresholds of 460 ms, 480 ms, and 500 ms is presented in Table 1. Generally, the Bazett formula produced more results of a QTc longer than 460 ms than the referral method. Prolongation of QTc ≥ 480 ms also more often occurred at the third month if the Bazett instead of the Fridericia formula was used (Table 1).

The use of the Bazett instead of the Fridericia formula seriously increased the odds of identifying QTc prolongation (Table 2). In general, the odds ratio for the identification of QTc ≥ 460 ms with the Bazett formula was 5.49 (95% CI 3.28–9.19) higher than with the Fridericia rule.

### 3.3. Prediction of QTcF Prolongation

To predict QTcF ≥ 460 ms at month 6, a logistic regression model was constructed.

A LASSO logistic regression model identified age, baseline QTcF, QTcF at month 3, and EF at month 3 as predictors of QTcF ≥ 460 ms at month 6 (Table 3). Increasing age was associated with higher odds of the outcome (β = 0.084, OR = 1.087 per year). Baseline QTcF and QTcF at month 3 were also positively associated with the risk of QTcF ≥ 460 ms at month 6 (β = 0.017, OR = 1.017 and β = 0.009, OR = 1.009, respectively, per 1 ms increase). In contrast, EF at month 3 was inversely associated with the outcome, with a 10-unit increase in EF corresponding to lower odds of QTcF ≥ 460 ms at month 6 (β = −0.199, OR = 0.819). The model intercept was β = −16.960. The selected predictors and corresponding regression coefficients and odds ratios are summarized in Table 3.

## 4. Discussion

Although this study demonstrates a relatively low incidence of significant QTc prolongation and absence of life-threatening arrhythmias in an ambulatory cohort free of preexisting cardiovascular disease, several considerations challenge the generalizability and underscore persistent risks. The exclusion of patients with known cardiovascular disorders strengthens attribution to therapies but limits applicability to real-world settings, where cancer patients often harbor cardiovascular risk factors, polypharmacy, and subclinical vulnerabilities despite no overt disease. Real-world data report QTc prolongation in up to 30% of patients on targeted antineoplastics—higher than in controlled cohorts—linked to concomitant medications like loop diuretics and variable monitoring [13,19,20].

Real-World Incidence: Significant QTc prolongation is relatively low in the ambulatory setting compared to hospitalized cancer patients [2,20,21]. This discrepancy likely reflects the higher burden of comorbid illnesses and profound electrolyte disturbances typically encountered in inpatient populations, which substantially exacerbate pro-arrhythmic risks [22]. Moreover, the observed transient fluctuations in QTc duration suggest that myocardial repolarization in cancer patients are highly individualized rather than strictly progressive, showing the need for serial electrocardiographic monitoring. Lasso logistic regression analysis showed that prior QTc results demonstrated a modest positive associations with risk of QTc increase, while age had a more important impact on that. Heatmap analysis indicates both temporal variability in repolarization parameters and differential effects across treatment classes, with the most consistent QTc prolongation signal observed in anthracycline-based therapies and selected targeted or platinum-based regimens. However, this pattern may be rather related to the larger number of patients that were treated with these compounds in the current study.

The observed variability in repolarization times across study time points shows that the Fridericia formula provides a more reliable QTc assessment by minimizing the rate-dependent bias inherent in Bazett’s method [23,24]. This is especially important in patients with cancer who experience tachycardia due to anemia, fever, or electrolyte disturbances.

Additionally, our findings corroborate existing evidence that, while some anticancer agents necessitate rigorous ECG surveillance, the absolute risk of life-threatening arrhythmias in stable, unselected oncology patients remains manageable [12,13,25]. Consequently, these results argue for a risk-stratified approach to cardiac monitoring, prioritizing patients with established cardiovascular comorbidities or those receiving multi-drug regimens known to interact with repolarization pathways [12,22]. Future clinical protocols should prioritize integrating real-world pharmacy prescription data to better account for potential pharmacodynamic interactions that may influence these electrocardiographic parameters [12]. Thus, the development of predictive models that incorporate both clinical risk factors and serialized ECG data might be essential for refining intervention thresholds in cardio-oncology practice. Furthermore, the Lasso logistic regression analysis that was performed shows that EF should be taken into account in those models as a protective factor for QTc elongation in contrast to age and preceding QTc results. Validating these models across diverse oncology cohorts will ensure that screening protocols remain both clinically effective and resource-efficient in the era of personalized cancer care [20].

Impact of Formula Choice: The importance of selecting an appropriate QTc correction formula was shown in this study, as different methods can yield varying baseline QTc values [26], potentially influencing the assessment of cardiotoxicity in cancer patient. Using the Fridericia formula may prevent unnecessary oncology treatment interruptions caused by the overestimation inherent in the Bazett formula [23,24,27,28,29].

Clinical Implications: Despite statistical increases in QTc, the low incidence of TdP-risk levels suggests many chemotherapy regimens are safer than previously feared regarding repolarization [7,30]. Regardless of the correction formula employed, the observed incidence of significant QTc prolongation (i.e., ≥60 ms increase from baseline or ≥500 ms) remained relatively low in this cohort, remaining in line with previous findings in ambulatory oncology settings.

Study limitations: This is a post hoc analysis of an observational study focused on echocardiographic changes. The analysis is based on the data from an original database. However, ECG tracings were not analyzed during this analysis and investigators used the originally imputed QT and heart rate values. The number of available ECG recordings decreased at subsequent follow-up time points, which may have affected the precision of the longitudinal analyses. However, this attrition did not appear to materially affect the observed differences between QTc correction formulas. Other potential confounding factors, including polypharmacy, supportive medications, and changes in serum electrolyte levels during oncological treatment, were not systematically assessed and could be considered in future analyses.

## 5. Conclusions

The study findings indicate that although baseline QTc values were generally within acceptable limits, a subset of patients experienced clinically significant QTc prolongation during treatment, necessitating continued vigilance and further research into predictive biomarkers for this adverse event. The importance of selecting an appropriate QTc correction formula is crucial to prevent unnecessary oncology treatment interruptions. Future research should therefore focus on identifying specific patient profiles and treatment regimens that predispose individuals to QTc prolongation, alongside developing more refined predictive models.

## Figures and Tables

**Table 1:** Incidence of QTc prolongation at different time points, according to the calculation method.

Timepoint	Method of QTc Calculation	Prolongation of QTc ≥ 460 ms	Prolongation of QTc ≥ 480 ms	Prolongation of QTc ≥ 500 ms
Baseline *N* = 291	QTcF	6 (2.1%) ****	4 (1.4%)	1 (0.3%)
	QTcB	29 (10%)	8 (2.7%)	5 (1.7%)
Month 3 *N* = 242	QTcF	4 (1.7%) ****	1 (0.4%) ***	1 (0.4%)
	QTcB	31 (12.8%)	13 (5.4%)	3 (1.2%)
Month 6 *N* = 167	QTcF	6 (3.6%) **	2 (1.2%)	0 (0%)
	QTcB	21 (12.6%)	6 (3.6%)	2 (1.2%)
Month 12 *N* = 128	QTcF	2 (1.6%) *	0 (0%)	0 (0%)
	QTcB	9 (7%)	1 (0.8%)	1 (0.8%)

Columns 3–5 show the number of cases with increased QTc out of all measurements available. QTcF—QTc measured using Fridericia method; QTcB—QTc measured using Bazett method. **** *p* < 0.0001 as compared with QTcB; *** *p* = 0.0016 as compared with QTcB; ** *p* < 0.0041 as compared with QTcB; * *p* < 0.0335 as compared with QTcB.

**Table 2:** Odds of exceeding predefined QTc thresholds with Bazett correction (QTcB) relative to Fridericia correction (QTcF) at different timepoints.

Time Point	OR (≥460 ms)	95% CI	OR (≥480 ms)	95% CI	OR (≥500 ms)	95% CI
Baseline	5.26	2.15–12.87	2.03	0.60–6.81	5.07	0.59–43.67
Month 3	8.74	3.04–25.17	13.68	1.78–105.43	3.03	0.31–29.29
Month 6	3.86	1.52–9.83	3.07	0.61–15.46	5.06	0.24–106.21
Month 12	4.76	1.01–22.51	3.02	0.12–74.92	3.02	0.12–74.92

Values are ORs with 95% confidence intervals (CI). Sample sizes per time point: baseline: *n* = 291; month 3; *n* = 242; month 6: *n* = 167; month 12: *n* = 128.

**Table 3:** Predictors selected by LASSO logistic regression for QTcF ≥ 460 ms at month 6.

Variable	β Coefficient	Odds Ratio (OR)	Unit of OR Interpretation
Age	0.084	1.087	per 1 year increase
Baseline QTcF	0.017	1.017	per 1 ms increase
QTcF at month 3	0.009	1.009	per 1 ms increase
EF at month 3	−0.199 *	0.819 *	per 10-unit increase

* β and OR for EF at month 3 are expressed per 10-unit increase in EF.

## Data Availability

The datasets used and/or analyzed during the current study are available from the corresponding author upon reasonable request.

## Abbreviations

The following abbreviations are used in this manuscript:

BMI	Body mass index
CI	Confidence interval
ECG	Electrocardiogram
EF	Ejection fraction
ESC	European Society of Cardiology
IQR	Interquartile range
LV	Left ventricular
OR	Odds ratio
QT	QT interval
QTc	Corrected QT interval
QTcB	QT interval corrected using Bazett’s formula
QTcF	QT interval corrected using Fridericia’s formula
PTS	patients
SD	Standard deviation
TdP	Torsades de pointes
TKI	Tyrosine kinase inhibitor
